# Resting-State fMRI Using Passband Balanced Steady-State Free Precession

**DOI:** 10.1371/journal.pone.0091075

**Published:** 2014-03-12

**Authors:** Joe S. Cheng, Patrick P. Gao, Iris Y. Zhou, Russell W. Chan, Queenie Chan, Henry K. Mak, Pek L. Khong, Ed X. Wu

**Affiliations:** 1 Laboratory of Biomedical Imaging and Signal Processing, The University of Hong Kong, Hong Kong SAR, China; 2 Department of Electrical and Electronic Engineering, The University of Hong Kong, Hong Kong SAR, China; 3 Department of Anatomy, The University of Hong Kong, Hong Kong SAR, China; 4 Department of Medicine, The University of Hong Kong, Hong Kong SAR, China; 5 Diagnostic Radiology, The University of Hong Kong, Hong Kong SAR, China; 6 Philips Healthcare, Hong Kong SAR, China; Hangzhou Normal University, China

## Abstract

**Objective:**

Resting-state functional MRI (rsfMRI) has been increasingly used for understanding brain functional architecture. To date, most rsfMRI studies have exploited blood oxygenation level-dependent (BOLD) contrast using gradient-echo (GE) echo planar imaging (EPI), which can suffer from image distortion and signal dropout due to magnetic susceptibility and inherent long echo time. In this study, the feasibility of passband balanced steady-state free precession (bSSFP) imaging for distortion-free and high-resolution rsfMRI was investigated.

**Methods:**

rsfMRI was performed in humans at 3 T and in rats at 7 T using bSSFP with short repetition time (TR = 4/2.5 ms respectively) in comparison with conventional GE-EPI. Resting-state networks (RSNs) were detected using independent component analysis.

**Results and Significance:**

RSNs derived from bSSFP images were shown to be spatially and spectrally comparable to those derived from GE-EPI images with considerable intra- and inter-subject reproducibility. High-resolution bSSFP images corresponded well to the anatomical images, with RSNs exquisitely co-localized to the gray matter. Furthermore, RSNs at areas of severe susceptibility such as human anterior prefrontal cortex and rat piriform cortex were proved accessible. These findings demonstrated for the first time that passband bSSFP approach can be a promising alternative to GE-EPI for rsfMRI. It offers distortion-free and high-resolution RSNs and is potentially suited for high field studies.

## Introduction

Resting-state functional MRI (rsfMRI) examines the temporal correlations in blood oxygenation level-dependent (BOLD) signal in the absence of stimulus or task. The two most common rsfMRI analysis methods are seed-based analysis and independent component analysis (ICA). While the seed-based analysis tests a specific hypothesis by cross-correlating voxel-wise rsfMRI data with the time course within a priori defined region of interest [1], ICA provides a hypothesis-free decomposition of the data into spatially maximally independent components [2]. Regardless of the technique, a consistent observation is that the coherent patterns of low frequency fluctuations (<0.1 Hz) in BOLD signal reflect a series of functional networks, the so-called resting-state networks (RSNs), in both humans [1], [3], [4] and animals [5], [6], [7]. Extensive efforts in this field provide new insights into both brain functional organization [8], [9] and functional reorganization during disease, aging and learning [10], [11], [12].

To date, most rsfMRI studies have been conducted with gradient-echo (GE) echo planar imaging (EPI), which provides a good compromise between spatial and temporal resolution. Although highly successful in most brain regions, this technique has limitations that reduce image quality in areas with high magnetic susceptibility. The requirement for long echo time (TE) to achieve BOLD sensitivity makes the technique susceptible to signal dropout [13], while the use of EPI for high speed can introduce severe image distortion [14]. Therefore, rsfMRI in brain regions near susceptibility boundaries, such as human anterior prefrontal cortex and rat piriform cortex, can be difficult. Additionally, high field improves the signal to noise ratio (SNR) and sensitivity of rsfMRI [15], but it also increases the field inhomogeneites and susceptibility effects that cause image distortion and signal dropout. To overcome these limitations, a variety of methods have been proposed, including parallel imaging [16], spiral-in/out acquisition [17], 3D z-shimming [18] and so on. However, each method has its own penalties such as decreased SNR, blurring with spiral readout or reduced temporal resolution. The other limiting factor in EPI approach is the significant T_2_*-weighting during the long readout that can cause warping and loss of spatial resolution [19], [20].

Balanced steady-state free precession (bSSFP) sequence is an imaging technique that uses rapid radiofrequency (RF) excitation pulses combined with fully balanced gradient pulses during each excitation repetition time (TR). Its short TR and readout duration, together with high SNR, have the potential for fast, distortion-free and high-resolution functional imaging. For a comprehensive review, see Miller [21]. The original bSSFP task-evoked fMRI studies used the steep magnitude/phase transition in the bSSFP off-resonance profile [22], [23], [24]. Despite its functional potential, the use of transition band bSSFP was limited by the need for multi-frequency acquisitions to locate the narrow range transition band. Later, it was demonstrated that functional contrast could also be achieved by using the relatively large flat portion of the bSSFP profile [25], [26], [27], [28], [29]. The contrast mechanism of this ‘passband bSSFP’ is largely similar to that of spin-echo (SE). Recent years have seen compelling applications of the passband bSSFP in distortion-free and high-resolution functional imaging, such as layer-specific fMRI to hypoxic challenges in the mouse retinal [30] and high-fidelity auditory tonotopy mapping [31]. A preliminary study also reported odor-evoked activity in the human olfactory bulb, a small structure in high-susceptibility region [32].

In this study, the feasibility of bSSFP rsfMRI was investigated in humans at 3 T and in rats at 7 T. RSNs derived from bSSFP images using ICA were compared with those derived from GE-EPI images. Intra- and inter-subject reproducibility of bSSFP rsfMRI was also examined. Moreover, RSNs of high-resolution bSSFP rsfMRI were evaluated with regard to their anatomical correspondence. Finally, distortion-free bSSFP rsfMRI was demonstrated in human anterior prefrontal cortex and rat piriform cortex.

## Methods

### Ethics Statement

The human study was approved by the institutional review board of the University of Hong Kong. The participants were informed of the aims of our study before MRI examinations. Full written informed consent was obtained from each participant. The animal study was approved by the Committee on the Use of Live Animals in Teaching and Research of the University of Hong Kong. All animal experiments were carried out in strict accordance with the recommendations in the Guidelines for the use of Experimental Animals of the University of Hong Kong.

### Human Imaging

Eleven healthy right-handed subjects (6 male and 5 female; age 23.3±2.8) participated in this study. MRI was performed on a Philips 3T MRI Achieva scanner (Philips Healthcare, Best, The Netherlands) with an 8-channel SENSE head coil. During rsfMRI, subjects were instructed to rest with their eyes open. bSSFP scan was performed with alternating RF pulse (β = π), number of slices (Nslices) = 1, slice thickness = 5 mm, field of view (FOV) = 240×240 mm2, matrix size = 64×64 (reconstructed to 128×128 by zero-filling), TR/TE = 4/2 ms, number of signal averaged (NSA) = 12 yielding a temporal resolution = 3 s and number of dynamic scans (Ndyn) = 100. An optimal flip angle (FA) of 30o was estimated with T_1_ = 1820 ms and T_2_ = 99 ms [33] by cos(FA_opt_) = (T_1_/T_2_-1)/(T_1_/T_2_+1) to provide the maximum flat passband region in the bSSFP off-resonance profile [34]. Volume shimming was performed prior to each bSSFP acquisition to avoid the narrow transition band and minimize banding artifacts. For comparison, GE-EPI scan was performed with TR/TE = 3000/30 ms, FA = 90°, SENSE factor = 2, Nslices = 33, slice thickness/gap = 5/0 mm, FOV = 240×240 mm^2^, matrix size = 64×64 (reconstructed to 128×128 by zero-filling) and Ndyn = 100. 2D T_1_-weighted (T_1_W) fast spin-echo (FSE) images were acquired for anatomical referencing, with TR/TE = 450/10 ms, FOV = 240×240 mm^2^, matrix size = 240×240 and slice thickness = 5 mm.

During each session, a whole-brain GE-EPI (Nslices = 33) scan was first performed. Based on the orthogonal scout images, the GE-EPI scan was positioned parallel to the anterior commissure - posterior commissure plane with the center slice covering the posterior cingulate cortex, and other slices covering intraparietal cortex, motor cortex, visual cortex and superior temporal cortex. Subsequent bSSFP (Nslices = 1) scans were performed with each scan matching its GE-EPI counterpart slice location. To evaluate the bSSFP rsfMRI approach, four experiments were designed as follows. First, to examine the feasibility of bSSFP rsfMRI, five bSSFP scans were acquired from one subject (Subject 1) within one session, with each scan covering the posterior cingulate cortex (PCC), intraparietal cortex, motor cortex, visual cortex and superior temporal cortex respectively, where RSNs were most commonly reported [35]. Second, to investigate intra-subject reproducibility, four more sessions were performed on different days on Subject 1 with a bSSFP scan covering the PCC in each session. To examine the inter-subject reproducibility, one session was performed on each of ten other subjects (Subjects 2–11) with a bSSFP scan covering the visual cortex in each session. Third, to examine its potential for high-resolution rsfMRI, bSSFP scans with matrix size = 128×128 covering the PCC were also acquired from Subjects 2–5 in the above-mentioned sessions. Temporal resolution of 3 s was maintained by SENSE acceleration (SENSE factor = 2) together with TR/TE = 4.8/2.4 ms and NSA = 15. Finally, to demonstrate the feasibility of bSSFP for distortion-free RSN detection, one session was performed on Subject 1 with a bSSFP scan covering the anterior prefrontal cortex (aPFC) near the air-tissue interface.

### Animal Imaging

Nine normal male Sprague-Dawley rats (200–250 g) were used in this study. Animals were initially anaesthetized with 3% isoflurane and then mechanically ventilated with 1.2–1.5% isoflurane via oral intubation. To minimize motion, animals were secured in the prone position on a plastic holder with the head fixed with a tooth bar and plastic screws in the ear canals. Animals were kept warm with circulating water while respiration rate, heart rate, oxygenation saturation and rectal temperature were continuously monitored and vital signs were maintained within normal physiological ranges [36], [37], [38].

MRI was performed on a 7 T Bruker scanner with a maximum gradient of 360 mT/m (70/16 PharmaScan, Bruker Biospin GmbH, Germany), a 72 mm birdcage transmit-only RF coil and an actively decoupled receive-only quadrature surface coil. Scout T_2_-weighted (T_2_W) images were first acquired in three planes with a rapid acquisition with relaxation enhancement (RARE) pulse sequence. For rsfMRI, bSSFP scans were acquired with alternating RF pulse (β = π), FA = 19° as optimized using T_1_ = 1754 ms and T_2_ = 45 ms [29], Nslices = 1, slice thickness = 1 mm, TR/TE = 2.506/1.253 ms, matrix size = 64×64 and NSA = 9 resulting in a temporal resolution = 1.5 s. Multiple bSSFP scans were performed on three animals with imaging planes covering primary visual cortex (VC) (Bregma – 7.2 mm) with FOV = 32×32 mm^2^, on five animals covering primary somatosensory cortex (SC) (Bregma – 3.2 mm) with FOV = 32×32 mm^2^ and on one animal covering piriform cortex (PC) (Bregma 3.3) with FOV = 26×26 mm^2^. Each resting-state scan had Ndyn of 300, lasting 7 minutes 30 seconds. For comparison, one GE-EPI scan with TR/TE = 1500/18 ms, FA = 30°, matrix size = 64×64, Nslices = 10 and slice thickness/gap = 1/0 mm was performed on each animal with one slice covering VC or SC. No resting-state GE-EPI scan was performed to cover PC due to the severe image distortion and signal dropout. Prior to rsfMRI scans local shimming was performed with a FieldMap based procedure [39] to avoid the narrow transition band and minimize banding artifacts in bSSFP. For anatomical referencing, 2D T_2_W RARE images were acquired at the same slice locations with TR/TE = 4200/36 ms, matrix size = 256×256, echo train length = 4 and NSA = 2.

### Data Preprocessing

The corresponding slices in a GE-EPI scan that matched any bSSFP imaging plane were extracted and treated as a single-slice scan prior to pre-processing and rsfMRI analysis. The first five volumes of each resting-state scan were discarded to avoid possible non-equilibrium effect in bSSFP and GE-EPI dynamic scans. For the human data, the following preprocessing steps were carried out using FSL v3.3 (FMRIB, Oxford, UK): motion correction [40], removal of nonbrain structures from GE-EPI using BET [41] and those from bSSFP using manually drawn masks, spatial smoothing using Gaussian kernel with full width half-maximum (FWHM) 5 mm and temporal band-pass filtering with 0.001–0.1 Hz cutoff. For the rat data, similar preprocessing steps were performed: intra-animal image realignment by 2D rigid-body transformation using AIR v5.2.5 (Roger Woods, UCLA, USA), spatial smoothing using Gaussian kernel with FWHM 0.5 mm and temporal band-pass FIR filtering with 0.005–0.1 Hz cutoff using MATLAB (MathWork, Natick, MA).

### Resting-state fMRI Analysis

Resting-state fMRI analysis was carried out using independent component analysis (ICA) on single-slice bSSFP and GE-EPI scans individually. ICA was implemented in MELODIC v3.1 [42] with 15 independent components (ICs) for the human data and in GIFT v2.0d (http://www.nitrc.org/projects/gift/) with 10 ICs for the rat data respectively. A mixing matrix was estimated in the ICA containing spatially ICs and corresponding time courses. The spatial IC was then converted to z-scores, which reflected the degree to which the time course of a given voxel correlated with that of the IC [43]. The histograms of IC z-scores were plotted and examined. The z-maps were thresholded based on a histogram mixture modeling at a probability (P-value) threshold >0.5 by using an alternative hypothesis-testing approach, placing equal loss to both false negatives and false positives (false positive rate = 0.5) [42], [44], [45]. While the thresholds differed in z-scores, they corresponded to the same probability. Statistical comparisons were carried out to examine the sensitivity of bSSFP for rsfMRI, the intra-subject and inter-subject reproducibility. The number of pixels with z-scores above the probability threshold and the mean z-scores of these pixels were statistically compared between the bSSFP and GE-EPI derived RSNs using two-tailed paired t-test with p<0.05 considered as significant. All the results were presented as means ± standard deviations.

To assess low frequency (<0.1 Hz) power distribution, each time course was converted to power spectrum using Fast Fourier Transform (FFT) after detrending. The power spectrum was normalized by dividing its values by its maximum.

RSNs were identified by examining each IC's spatial location and power spectrum, which should be localized in anatomically meaningful structures and dominated by low frequency [46]. Interpretations of RSNs were achieved with reference to previous ICA studies [47], [48] in conjunction with atlases for human brain (Harvard-Oxford cortical structural atlas, http://www.cma.mgh.harvard.edu/) and rat brain [49].

## Results

### Human Data

**Figure 1:**
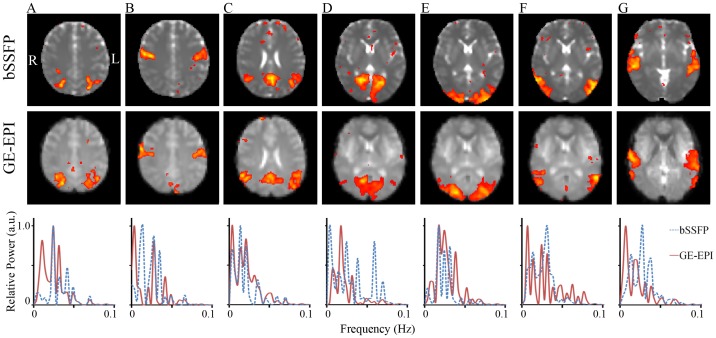
Resting-state networks (RSNs) of human derived from bSSFP and GE-EPI images. RSNs identified representing dorsal attention, primary motor cortex, posterior part of default mode network, occipital visual cortex (VC), medial VC, lateral VC and auditory cortex (A–G) using independent component analysis. bSSFP and GE-EPI images were acquired from the same subject with identical temporal resolution, spatial geometry and resting-state paradigm. Power spectra, converted from time courses of corresponding independent components using Fast Fourier Transform, clearly indicate low frequency dominance. Z-maps were thresholded using histogram mixture modeling at an alternative hypothesis threshold P>0.5.

Figure 1 illustrates the seven RSNs derived from bSSFP images and their GE-EPI counterparts acquired from the same subject at identical slice locations, with the former showing a close spatial and spectral resemblance to the latter, as well as the typical, well-known RSNs demonstrated by numerous rsfMRI studies (For an overview see: Smith et al., 2009). Fig. 1A shows the intraparietal sulcus, known to be part of the dorsal attention network [50]. Fig. 1B illustrates the precentral gyrus, or the primary motor cortex (MC). Fig. 1C shows the posterior cingulate cortex (PCC) and the lateral parietal cortex, posterior part of a regular default mode network. Figs. 1D–F represent the visual networks split into occipital, medial and lateral visual cortices (VCs). Fig. 1G shows the superior temporal cortex from the auditory network. The power spectra of all of the above RSNs, derived from both bSSFP and GE-EPI, clearly exhibited low frequency dominance.

**Figure 2:**
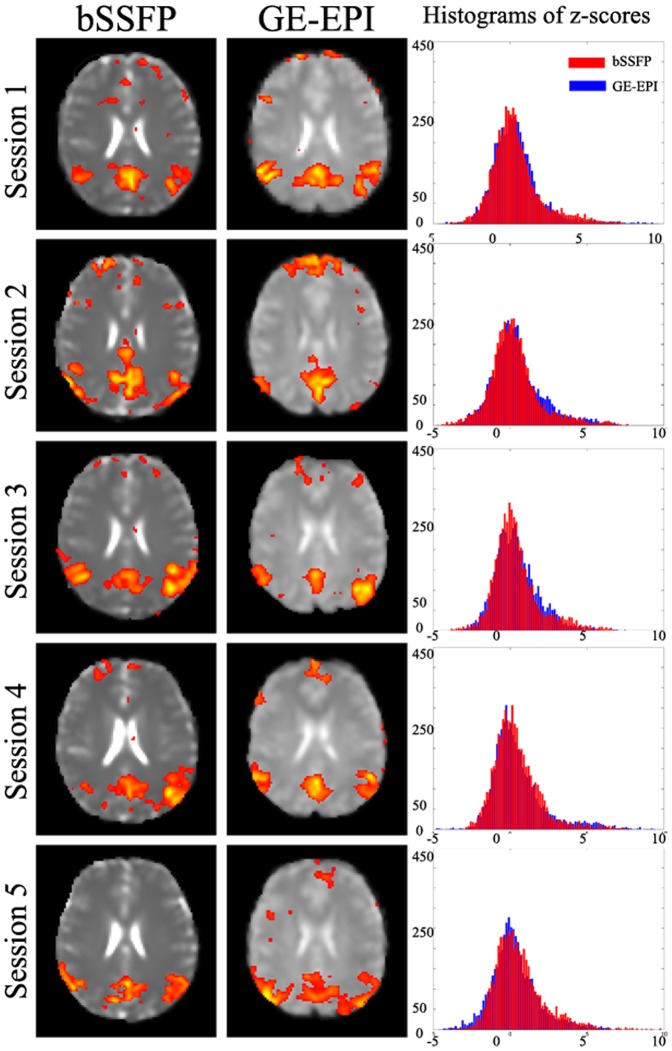
Intra-subject reproducibility of bSSFP for rsfMRI. RSN representing default mode network was consistently identified from bSSFP and GE-EPI scans acquired from one subject at five sessions. Z-scores were plotted into histograms, showing clear Gaussian distributions. Z-maps were thresholded using histogram mixture modeling at an alternative hypothesis threshold P>0.5.

**Figure 3:**
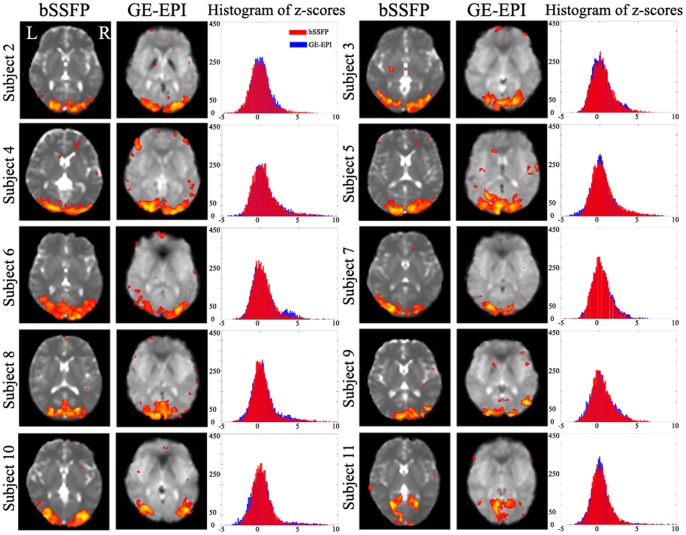
Inter-subject reproducibility of bSSFP for rsfMRI. RSN representing visual cortex was consistently identified from bSSFP and GE-EPI scans acquired from ten subjects. Z-scores were plotted into histograms, showing clear Gaussian distributions. Z-maps were thresholded using histogram mixture modeling at an alternative hypothesis threshold P>0.5.

The intra-subject reproducibility of bSSFP rsfMRI is demonstrated in Figure 2. Spatial patterns of one RSN of the same subject examined in five sessions on different days were found similar. To examine the inter-subject reproducibility, resting-state bSSFP and GE-EPI scans were collected from ten subjects. Figure 3 shows that similar RSNs for visual cortex were consistently identified across all the ten subjects. Furthermore, z-scores of all RSNs derived from bSSFP and GE-EPI images were plotted into histograms, showing clear Gaussian distributions. Based on a histogram Gaussian/Gamma mixture modeling, each z-map was thresholded at the same probability threshold (P>0.5). No significant difference was detected in the number of pixels and mean z-scores above thresholds between the RSNs derived from the two imaging methods (Table 1).

**Table 1:** bSSFP and GE-EPI rsfMRI reproducibility.

	bSSFP	GE-EPI	P
Intra-subject reproducibility (1 subject with 5 sessions)			
Mean z	3.79±0.35	3.85±0.08	0.30
Number of pixels	530±119	560±82	0.75
Inter-subject reproducibility (10 subjects with single session)			
Mean z	3.73±0.59	3.97±0.48	0.28
Number of pixels	585±161	475±111	0.10

Means and standard deviations of the number of pixels with z-scores above the probability threshold (P>0.5) and the mean z-scores of these pixels in RSNs in Figures 2 and 3. Statistical analysis was performed using two-tailed paired t-test.

**Figure 4:**
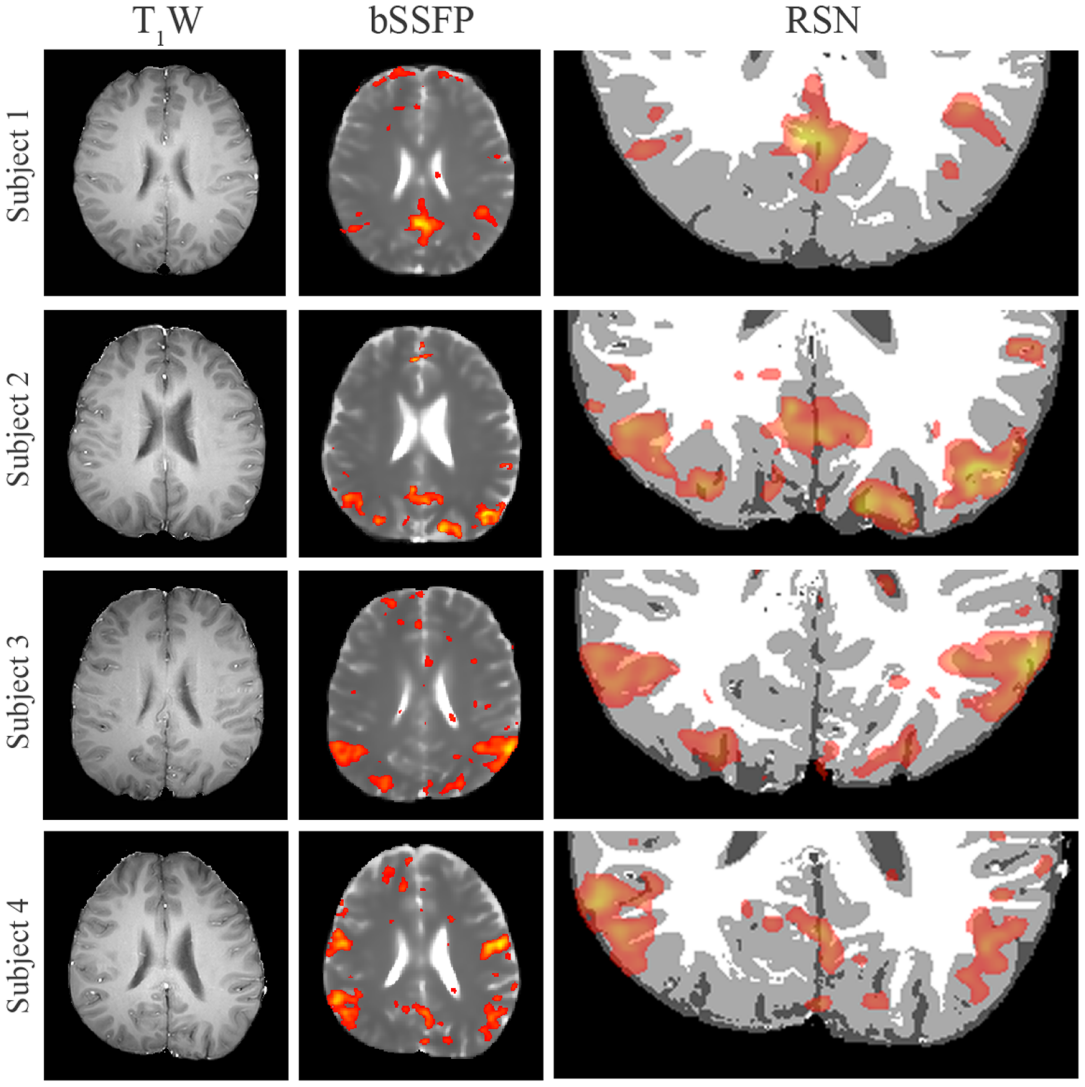
High-resolution RSNs derived from bSSFP images. T_1_W FSE anatomical references, bSSFP images at high-resolution (in-plane resolution 1.88×1.88 mm^2^, matrix size = 128×128, reconstructed to 240×240 by zero-filling) and zoomed and segmented brain images. High-resolution bSSFP images well corresponded to their T_1_W references, with RSNs exquisitely co-localized to the gray matter without any co-registration. T_1_W images were segmented into white matter, gray matter and cerebrospinal fluid.

High-resolution bSSFP images were also acquired at in-plane resolution of 1.88×1.88 mm^2^ (matrix size = 128×128, reconstructed to 240×240 by zero-filling). As shown in Figure 4, high-resolution bSSFP (middle column) images corresponded well to the T_1_W FSE images (left column). Furthermore, without any co-registration the RSNs derived (right column) exquisitely co-localized to the gray matter segmented from T_1_W images using FAST [51].

**Figure 5:**
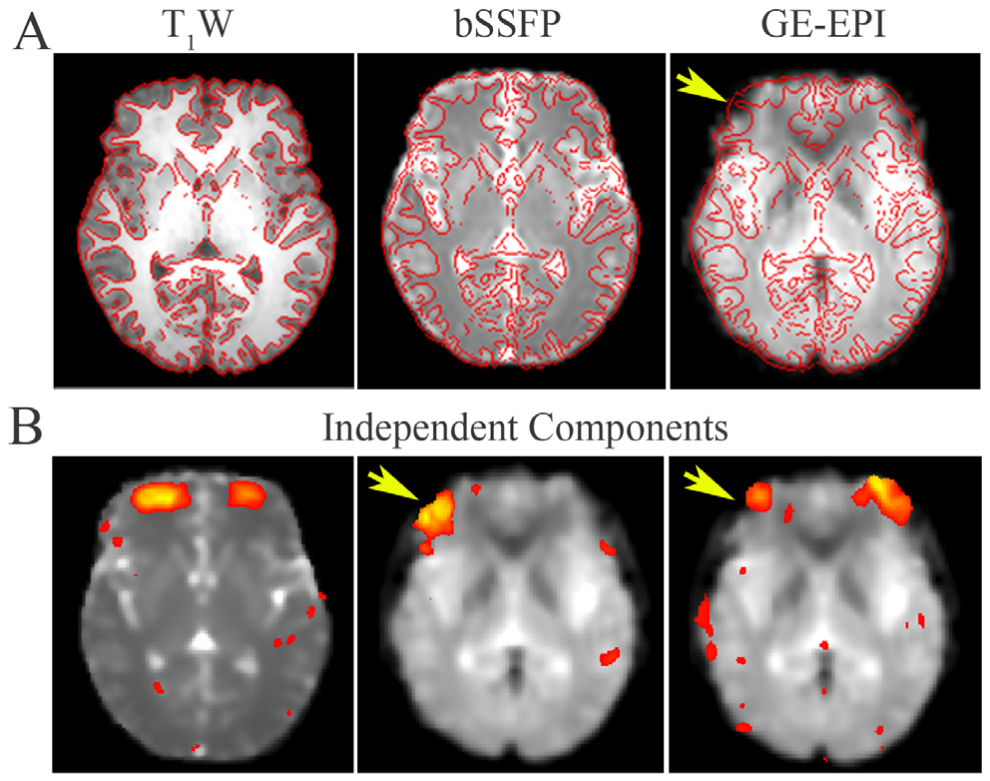
A distortion-free RSN derived from bSSFP images at human anterior prefrontal cortex (aPFC). **A**: T_1_W FSE, bSSFP and GE-EPI images at identical slice location were overlaid by edges of T_1_W images detected using FSL-slicer tool. Image distortion and signal dropout in GE-EPI (SENSE factor = 2) were pointed by an arrow head, while bSSFP image showed excellent overlap with T_1_W images. **B**: Independent components derived from bSSFP (left) and GE-EPI (middle and right) situated at aPFC. Note that the clusters within the latter two were adjacent to image distortion and signal dropout, affecting the precise interpretation of their neurophysiological significance.

Figure 5 illustrates the distortion-free RSN mapping capability of bSSFP. The anterior prefrontal cortex (aPFC) was imaged by T_1_W FSE, bSSFP and GE-EPI sequences with identical geometry. All images were overlaid with edges of the T_1_W reference (Fig. 5A). At aPFC, image distortion and signal dropout were obvious in the GE-EPI image even with SENSE factor = 2 (as pointed by arrows in Fig. 5A). The bSSFP image, however, showed an excellent overlap with the T_1_W reference. A RSN representing aPFC was detected from bSSFP (Fig. 5B, left). In contrast, of all those derived from GE-EPI two independent components were found located at aPFC. They were considered, however, unreliable because they bordered on the regions suffered from severe image distortion and signal dropout (Fig. 5B, middle and right).

### Animal Data

**Figure 6:**
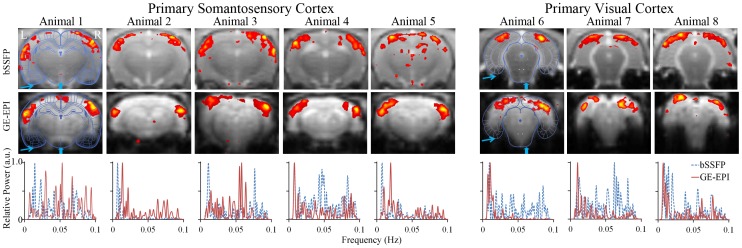
RSNs of rats derived from bSSFP and GE-EPI images. Consistent RSNs including primary somatosensory cortex (SC) and primary visual cortex (VC) across animals were obtained from both bSSFP and GE-EPI images with individual power spectra dominated by very low frequency (<0.04 Hz). SC, Bregma - 3.2 mm; VC, Bregma - 7.2 mm.

bSSFP approach for rsfMRI was performed and compared with GE-EPI in isoflurane-anesthetized rats. As shown in Figure 6, robust RSNs were derived from bSSFP and GE-EPI images covering SC from all the five animals and covering VC from all the three animals. The spatial patterns of each RSN were consistent across animals and largely comparable between the two approaches. Moreover, the two spectra yielded by them were both low frequency dominated. High power in previously reported low frequency range (below 0.04 Hz) [52] was observed in most rats, with occasional appearance of peaks between 0.04 Hz and 0.1 Hz. It should also be noted that GE-EPI images were affected by susceptibility artifacts at lower brain areas close to the ear canal and airway (as pointed by arrows in Fig. 6), whereas bSSFP images were in good agreement with rat brain atlas [49].

**Figure 7:**
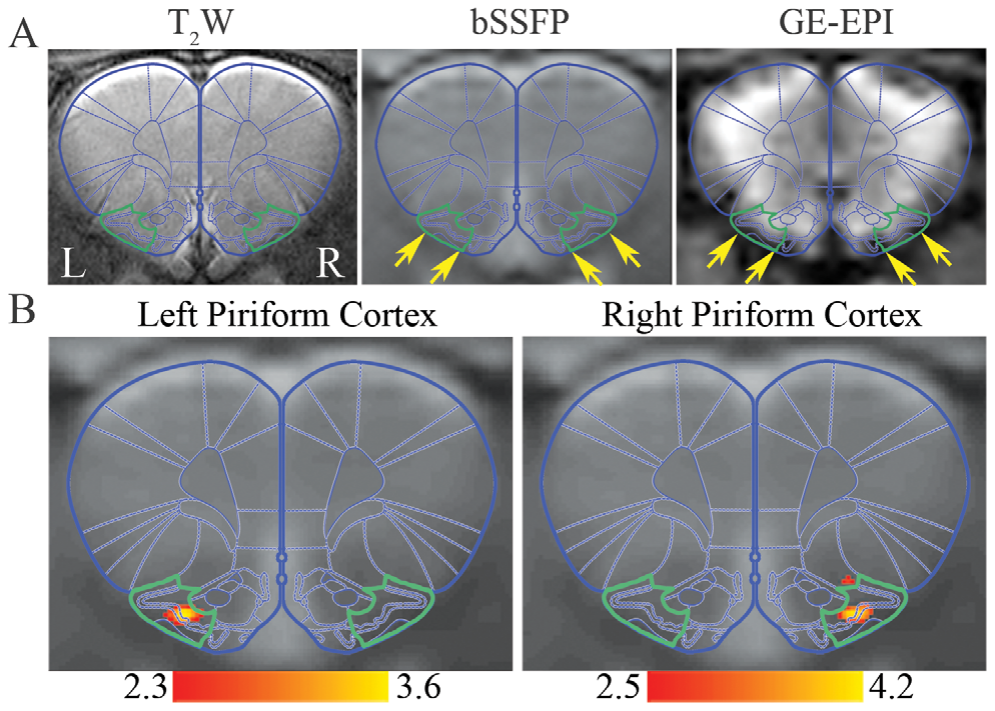
A distortion-free RSN derived from bSSFP images at rat piriform cortex (PC). **A**: T_2_W RARE, bSSFP and GE-EPI images at identical slice location covering PC. PCs were overlaid by green contours based on atlas [49]. Note that GE-EPI suffered from image distortion and signal dropout at inferior and lateral PCs as pointed by arrow heads, while bSSFP was in good agreement with T_2_W image. **B**: Unilateral RSNs for left and right PCs derived from bSSFP. PC, Bregma 3.3 mm.

Distortion-free RSN mapping capability of bSSFP was also investigated at the piriform cortex (PC) or the primary olfactory cortex, which is for one of the most important sensory functions in rats [53]. Figure 7A shows the typical T_2_W RARE, bSSFP and GE-EPI images at identical slice location of one animal, with PCs outlined in green based on the atlas. Image distortion and signal dropout at the inferior and lateral PCs were observed in GE-EPI image (as pointed by arrows in Fig. 7A, right). Since PCs are embedded with rich macrovasculature such as rostral rhinal veins [54], the image distortion and signal dropout were likely due to the susceptibility effects near large vessels in addition to brain tissue boundary. bSSFP images were, however, free of such susceptibility artifacts (Fig. 7A middle) and two unilateral RSNs for left and right PCs were identified (Fig. 7B).

## Discussion

### Feasibility of passband bSSFP rsfMRI

As demonstrated in this study, passband bSSFP sequence is a feasible alternative to GE-EPI for rsfMRI. As shown in Figs. 1, 2, 3 and 6 and Table 1, RSNs derived from bSSFP and GE-EPI images were essentially similar, both in close resemblance to the typical, well-known RSNs reported by numerous rsfMRI studies [47], [55]. Considerable intra- and inter-subject reproducibility was also observed (Figs. 2, 3 and Table 1), indicating the robustness of the proposed bSSFP approach for rsfMRI.

The resemblance in RSNs derived from bSSFP and GE-EPI images likely stems from the BOLD sensitivity common to both methods. Comparing to GE-EPI, the exact contrast mechanism of bSSFP remains elusive. It depends on the protocol parameters, particularly TR and FA [56], [57]. By employing large FA, bSSFP is optimized for the passband; while by employing small FA, bSSFP is optimized for the transition band. At relatively large FA (30°/19°) and short TR (4.0/2.5 ms) used in the current human and rat studies respectively, the off-resonance effects around microvasculature created by deoxyhemoglobin are in the same spatial scale of water diffusion distance and therefore the dynamic averaging effect cannot be refocused, leading to signal increase during local neuronal activation. This mechanism can be considered similar to that of T_2_W spin echo (SE). The main difference from SE is that bSSFP sequence allows for stimulated echo pathways that can give rise to increase in signal and BOLD contrast. bSSFP signal may also include an intravascular component of macrovasculature, mainly due to water molecules diffusion in and out of the red blood cells [25]. bSSFP with lengthened TR for high spatial resolution exhibits T_2_* dephasing and the sequence behaves essentially like a poorly-spoiled GE [58]. Therefore, the T_2_*W also contributed to the BOLD sensitivity in the present study of high-resolution bSSFP rsfMRI.

The above bSSFP contrast mechanism was established in the context of task-evoked responses. Although much is known about the neurophysiology underlying task-evoked BOLD responses [59], the neurophysiological basis of spontaneous modulation of BOLD signal remains to be debated. For example, nitric oxide synthase inhibition blocks task-evoked blood flow increases, but spontaneous fluctuations in blood flow can remain unaffected [60]. Nevertheless, it has been shown that both spontaneous and task-evoked GE-EPI BOLD responses depend on T_2_* BOLD contrast [61]. Hence, the spontaneous modulation of bSSFP signal most likely depends on T_2_ contrast, the same as that of bSSFP task-evoked responses. In addition, the power spectra of RSNs derived from both bSSFP and GE-EPI were shown to be dominated by low frequency, indicating that there exists a universal frequency signature of the spontaneous modulation of BOLD signal at resting-state.

Sensitivity of passband bSSFP rsfMRI is expected to be dependent of acquisition parameters. Parameters such as TR, TE and FA can influence the contrast mechanism by which the sensitivity changes. Changes in partial volume effects at different spatial resolution are also expected to impact sensitivity. However, when selecting the acquisition parameters the significance of the above effects has to be weighed against other constraints such as the flatness of passband, the width of passband (typically 0.75×TR^−1^ Hz) and RF specific absorption rate. In the current study with very short TR and optimized large FA, no significant difference was detected in the number of pixels and mean z-scores above thresholds between the RSNs derived from bSSFP and GE-EPI data. Moreover, at submillimeter resolution most RSNs derived from bSSFP exhibited more focal clusters than those from GE-EPI with rapidly varying z-scores among adjacent pixels (Fig. 6). Since bSSFP functional contrast is composed of not only the extravascular component sensitive to microvasculature but also the undesirable intravascular component sensitive to macrovasculature [57], the focal clusters in bSSFP derived RSNs maps might be due to partial volume effects from large veins. To minimize such undesirable effects, slight diffusion weighting or inversion recovery can be combined with bSSFP acquisition [30]. Nevertheless, it remains imperative to systematically investigate the sensitivity of passband bSSFP for rsfMRI in the future studies.

### High-resolution and Distortion-free RSN mapping

In the presence of signal dropout and image distortion, even with high encoding resolution, the effective spatial resolution can still be greatly reduced in GE-EPI with neighboring voxels inaccurately assigned in the image space. Furthermore, high acquisition resolution lengthens the readout and can further distort the image. While In passband bSSFP, the rapid excitation repetition intervals allow short segmented readouts, resulting in images with distortion levels similar to that of anatomical acquisitions (see human aPFC and rat PC images acquired with GE-EPI and bSSFP in Figs. 5 and 7A). Therefore, RSNs derived from bSSFP can be interpreted for functional connectivity with high fidelity. As shown in Fig. 4, RSNs derived from high-resolution bSSFP (1.88×1.88 mm^2^) exquisitely co-localized to the gray matter without any co-registration (Fig. 4). bSSFP also detected a RSN at aPFC (Fig. 5B), which has not been reported by GE-EPI at this slice location likely due to image distortion and signal dropout. Note that such a pattern has been detected by a recent SE-EPI study at 7 T [62]. Nevertheless, bSSFP approach offers advantages over SE-EPI approach for rsfMRI. It yields less image distortion with reduced readout, and it may also provide higher sensitivity, with higher signal and BOLD contrast arising from the stimulated echo pathways [27], [29]. In our rat results at 7 T, two unilateral components localized in PC were derived from bSSFP (Fig. 7). Bilateral PC RSNs have been reported in previous rat studies using GE-EPI, in which image distortion and signal dropout were alleviated by lower field strength at 4.7 T [5] or parallel imaging technique [6]. The absence of unilateral components in those studies may be caused by ICA underestimation and/or a high degree of temporal correlation between intra- and interhemispheric network. Homologous regions have been known to operate both uni- and bilaterally [63]. Note that unilateral components representing other sensory networks have been reported in somatosensory and motor cortices in rats and mice [6], [47].

While various methods have been proposed to reduce signal dropout and image distortion with conventional GE-EPI [16], [17], [18], [64], most of such approaches can be adopted for bSSFP imaging. Moreover, the inherently distortion-free nature of bSSFP circumvents the additional processes in both acquisition and post-processing that are often employed to tackle the image distortion problem.

### Technical Considerations

First, bSSFP requires good shimming to avoid banding artifacts particularly at high field. In this study, localized shimming was carefully performed within the single image slab prior to resting-state acquisition to minimize the banding artifacts [29]. Second, this study demonstrated the bSSFP rsfMRI with single slice acquisition. The differences in RSNs (medial VC for Subjects 2–9, lateral VC for Subject 10 and occipital VC for Subject 11 shown in Figure 3) could be caused by slightly different slice locations besides inter-subject variations in functional connectivity. However, fast 3D volumetric bSSFP fMRI has been demonstrated using various 3D k-space trajectories [22], [65] and with SENSE acceleration [66]. Such 3D acquisition can be readily adopted for rsfMRI. To cover the whole brain, a previous bSSFP fMRI study combined two acquisitions with different phase cycling angles to remove the banding artifacts [27]. Yet, recent fMRI studies have simulated [67] and implemented [68] a frequency-modulated bSSFP technique [69] enabling dynamic acquisition with different phase cycling angles without reestablishing the steady-state. This frequency-modulated technique could enable the bSSFP rsfMRI to measure temporal correlations within full brain. Combining such frequency-modulated technique, parallel imaging and/or compressed sensing and 3D acquisition, high spatial and temporal resolution bSSFP with full brain coverage can be achieved for rsfMRI.

## Conclusion

This study shows that passband bSSFP is feasible for rsfMRI studies free of severe image distortion and signal dropout. RSNs derived from bSSFP images were shown spatially and spectrally comparable to those derived from GE-EPI images with considerable intra- and inter-subject reproducibility in humans at 3 T and in rats at 7 T. High-resolution bSSFP corresponded well to the anatomical images, with RSNs exquisitely co-localized to gray matter. Furthermore, RSNs at areas of severe susceptibility were proved accessible including human anterior prefrontal cortex and rat piriform cortex. These findings demonstrated for the first time that passband bSSFP approach can be a promising alternative to GE-EPI for rsfMRI. It offers distortion-free and high-resolution RSNs and is potentially suited for high field studies.

## References

[1] Biswal B, Yetkin FZ, Haughton VM, Hyde JS (1995) Functional connectivity in the motor cortex of resting human brain using echo-planar MRI. Magnetic Resonance in Medicine 34: 537–541. 8524021 10.1002/mrm.1910340409

[2] Beckmann CF, Smith SM (2005) Tensorial extensions of independent component analysis for multisubject FMRI analysis. NeuroImage 25: 294–311. 15734364 10.1016/j.neuroimage.2004.10.043

[3] Fox MD, Raichle ME (2007) Spontaneous fluctuations in brain activity observed with functional magnetic resonance imaging. Nature Reviews Neuroscience 8: 700–711. 17704812 10.1038/nrn2201

[4] Cordes D, Haughton VM, Arfanakis K, Carew JD, Turski PA, et al (2001) Frequencies contributing to functional connectivity in the cerebral cortex in “resting-state” data. American Journal of Neuroradiology 22: 1326–1333. 11498421 PMC7975218

[5] Liang ZF, King JA, Zhang NY (2011) Uncovering Intrinsic Connectional Architecture of Functional Networks in Awake Rat Brain. Journal of Neuroscience 31: 3776–3783. 21389232 10.1523/JNEUROSCI.4557-10.2011 PMC3073070

[6] Jonckers E, Van Audekerke J, De Visscher G, Van der Linden A, Verhoye M (2011) Functional connectivity fMRI of the rodent brain: comparison of functional connectivity networks in rat and mouse. PLoS One 6: e18876. 21533116 10.1371/journal.pone.0018876 PMC3078931

[7] Lu H, Zuo Y, Gu H, Waltz JA, Zhan W, et al (2007) Synchronized delta oscillations correlate with the resting-state functional MRI signal. Proceedings of the National Academy of Sciences of the United States of America 104: 18265–18269. 17991778 10.1073/pnas.0705791104 PMC2084331

[8] Bullmore E, Sporns O (2009) Complex brain networks: graph theoretical analysis of structural and functional systems. Nature Reviews Neuroscience 10: 186–198. 19190637 10.1038/nrn2575

[9] Mueller S, Wang D, Fox MD, Yeo BT, Sepulcre J, et al (2013) Individual variability in functional connectivity architecture of the human brain. Neuron 77: 586–595. 23395382 10.1016/j.neuron.2012.12.028 PMC3746075

[10] Greicius MD, Srivastava G, Reiss AL, Menon V (2004) Default-mode network activity distinguishes Alzheimer's disease from healthy aging: evidence from functional MRI. Proceedings of the National Academy of Sciences of the United States of America 101: 4637–4642. 15070770 10.1073/pnas.0308627101 PMC384799

[11] Taubert M, Lohmann G, Margulies DS, Villringer A, Ragert P (2011) Long-term effects of motor training on resting-state networks and underlying brain structure. Neuroimage 57: 1492–1498. 21672633 10.1016/j.neuroimage.2011.05.078

[12] Jafri MJ, Pearlson GD, Stevens M, Calhoun VD (2008) A method for functional network connectivity among spatially independent resting-state components in schizophrenia. Neuroimage 39: 1666–1681. 18082428 10.1016/j.neuroimage.2007.11.001 PMC3164840

[13] Ojemann JG, Akbudak E, Snyder AZ, McKinstry RC, Raichle ME, et al (1997) Anatomic localization and quantitative analysis of gradient refocused echo-planar fMRI susceptibility artifacts. NeuroImage 6: 156–167. 9344820 10.1006/nimg.1997.0289

[14] Jezzard P, Clare S (1999) Sources of distortion in functional MRI data. Human Brain Mapping 8: 80–85. 10524596 10.1002/(SICI)1097-0193(1999)8:2/3<80::AID-HBM2>3.0.CO;2-C PMC6873315

[15] Smith SM (2012) The future of FMRI connectivity. NeuroImage 62: 1257–1266. 22248579 10.1016/j.neuroimage.2012.01.022

[16] de Zwart JA, van Gelderen P, Golay X, Ikonomidou VN, Duyn JH (2006) Accelerated parallel imaging for functional imaging of the human brain. NMR in Biomedicine 19: 342–351. 16705634 10.1002/nbm.1043

[17] Glover GH, Thomason ME (2004) Improved combination of spiral-in/out images for BOLD fMRI. Magnetic Resonance in Medicine 51: 863–868. 15065263 10.1002/mrm.20016

[18] Glover GH (1999) 3D z-shim method for reduction of susceptibility effects in BOLD fMRI. Magnetic Resonance in Medicine 42: 290–299. 10440954 10.1002/(sici)1522-2594(199908)42:2<290::aid-mrm11>3.0.co;2-n

[19] Johnson G, Hutchison JMS (1985) The limitations of NMR recalled-echo imaging techniques. Journal of Magnetic Resonance (1969) 63: 14–30.

[20] Farzaneh F, Riederer SJ, Pelc NJ (1990) Analysis of T2 limitations and off-resonance effects on spatial resolution and artifacts in echo-planar imaging. Magnetic Resonance in Medicine 14: 123–139. 2352469 10.1002/mrm.1910140112

[21] Miller KL (2012) FMRI using balanced steady-state free precession (SSFP). NeuroImage 62: 713–719. 22036996 10.1016/j.neuroimage.2011.10.040 PMC3398389

[22] Miller KL, Smith SM, Jezzard P, Pauly JM (2006) High-resolution FMRI at 1.5T using balanced SSFP. Magnetic Resonance in Medicine 55: 161–170. 16345040 10.1002/mrm.20753

[23] Miller KL, Hargreaves BA, Lee J, Ress D, DeCharms RC, et al (2003) Functional brain imaging using a blood oxygenation sensitive steady state. Magnetic Resonance in Medicine 50: 675–683. 14523951 10.1002/mrm.10602

[24] Scheffler K, Seifritz E, Bilecen D, Venkatesan R, Hennig J, et al (2001) Detection of BOLD changes by means of a frequency-sensitive trueFISP technique: Preliminary results. NMR in Biomedicine 14: 490–496. 11746942 10.1002/nbm.726

[25] Dharmakumar R, Hong J, Brittain JH, Plewes DB, Wright GA (2005) Oxygen-sensitive contrast in blood for steady-state free precession imaging. Magnetic Resonance in Medicine 53: 574–583. 15723410 10.1002/mrm.20393

[26] Miller KL, Smith SM, Jezzard P, Wiggins GC, Wiggins CJ (2007) Signal and noise characteristics of SSFP FMRI: A comparison with GRE at multiple field strengths. NeuroImage 37: 1227–1236. 17706432 10.1016/j.neuroimage.2007.06.024

[27] Lee JH, Dumoulin SO, Saritas EU, Glover GH, Wandell BA, et al (2008) Full-brain coverage and high-resolution imaging capabilities of passband b-SSFP fMRI at 3T. Magnetic Resonance in Medicine 59: 1099–1110. 18421687 10.1002/mrm.21576 PMC2694041

[28] Bowen CV, Menon RS, Gati JS (2005) High field balanced-SSFP fMRI: A BOLD technique with excellent tissue sensitivity and superior large vessel suppression. Proceedings of International Society of Magnetic Resonance in Medicine. pp. 119.

[29] Zhou IY, Cheung MM, Lau C, Chan KC, Wu EX (2012) Balanced steady-state free precession fMRI with intravascular susceptibility contrast agent. Magnetic Resonance in Medicine 68: 65–73. 22127794 10.1002/mrm.23202

[30] Duong TQ, Pardue MT, Thule PM, Olson DE, Cheng H, et al (2008) Layer-specific anatomical, physiological and functional MRI of the retina. NMR Biomed 21: 978–996. 18792422 10.1002/nbm.1311 PMC2752861

[31] Cheung MM, Lau C, Zhou IY, Chan KC, Zhang JW, et al (2012) High fidelity tonotopic mapping using swept source functional magnetic resonance imaging. Neuroimage 61: 978–986. 22445952 10.1016/j.neuroimage.2012.03.031

[32] Parrish T, Chen Y, Li W, Howard J, Gottfried J (2008) High resolution 3D functional images of the human olfactory bulb using passband SSFP at 3T. Proceedings of International Society of Magnetic Resonance in Medicine. pp. 3567.

[33] Stanisz GJ, Odrobina EE, Pun J, Escaravage M, Graham SJ, et al (2005) T-1, T-2 relaxation and magnetization transfer in tissue at 3T. Magnetic Resonance in Medicine 54: 507–512. 16086319 10.1002/mrm.20605

[34] Scheffler K, Lehnhardt S (2003) Principles and applications of balanced SSFP techniques. European radiology 13: 2409–2418. 12928954 10.1007/s00330-003-1957-x

[35] Damoiseaux JS, Rombouts SA, Barkhof F, Scheltens P, Stam CJ, et al (2006) Consistent resting-state networks across healthy subjects. Proceedings of the National Academy of Sciences of the United States of America 103: 13848–13853. 16945915 10.1073/pnas.0601417103 PMC1564249

[36] Cheung MM, Lau C, Zhou IY, Chan KC, Cheng JS, et al (2012) BOLD fMRI investigation of the rat auditory pathway and tonotopic organization. NeuroImage 60: 1205–1211. 22297205 10.1016/j.neuroimage.2012.01.087

[37] Chan KC, Xing KK, Cheung MM, Zhou IY, Wu EX (2010) Functional MRI of postnatal visual development in normal and hypoxic-ischemic-injured superior colliculi. NeuroImage 49: 2013–2020. 19879366 10.1016/j.neuroimage.2009.10.069

[38] Lau C, Zhang JW, Xing KK, Zhou IY, Cheung MM, et al (2011) BOLD responses in the superior colliculus and lateral geniculate nucleus of the rat viewing an apparent motion stimulus. NeuroImage 58: 878–884. 21741483 10.1016/j.neuroimage.2011.06.055

[39] Webb P, Macovski A (1991) Rapid, fully automatic, arbitrary-volume in vivo shimming. Magnetic Resonance in Medicine 20: 113–122. 1943653 10.1002/mrm.1910200112

[40] Jenkinson M, Bannister P, Brady M, Smith S (2002) Improved optimization for the robust and accurate linear registration and motion correction of brain images. NeuroImage 17: 825–841. 12377157 10.1016/s1053-8119(02)91132-8

[41] Smith SM (2002) Fast robust automated brain extraction. Human Brain Mapping 17: 143–155. 12391568 10.1002/hbm.10062 PMC6871816

[42] Beckmann CF, Smith SM (2004) Probabilistic independent component analysis for functional magnetic resonance imaging. IEEE Transactions on Medical Imaging 23: 137–152. 14964560 10.1109/TMI.2003.822821

[43] Greicius MD, Flores BH, Menon V, Glover GH, Solvason HB, et al (2007) Resting-state functional connectivity in major depression: Abnormally increased contributions from subgenual cingulate cortex and thalamus. Biological psychiatry 62: 429–437. 17210143 10.1016/j.biopsych.2006.09.020 PMC2001244

[44] De Luca M, Beckmann CF, De Stefano N, Matthews PM, Smith SM (2006) fMRI resting state networks define distinct modes of long-distance interactions in the human brain. NeuroImage 29: 1359–1367. 16260155 10.1016/j.neuroimage.2005.08.035

[45] Bullmore E, Brammer M, Williams SC, Rabe-Hesketh S, Janot N, et al (1996) Statistical methods of estimation and inference for functional MR image analysis. Magnetic Resonance in Medicine 35: 261–277. 8622592 10.1002/mrm.1910350219

[46] Cordes D, Haughton VM, Arfanakis K, Wendt GJ, Turski PA, et al (2000) Mapping functionally related regions of brain with functional connectivity MR imaging. American Journal of Neuroradiology 21: 1636–1644. 11039342 PMC8174861

[47] Hutchison RM, Mirsattari SM, Jones CK, Gati JS, Leung LS (2010) Functional networks in the anesthetized rat brain revealed by independent component analysis of resting-state fMRI. Journal of Neurophysiology 103: 3398–3406. 20410359 10.1152/jn.00141.2010

[48] Biswal BB, Mennes M, Zuo XN, Gohel S, Kelly C, et al (2010) Toward discovery science of human brain function. Proceedings of the National Academy of Sciences of the United States of America 107: 4734–4739. 20176931 10.1073/pnas.0911855107 PMC2842060

[49] Paxinos G, Watson C (2007) The rat brain in stereotaxic coordinates (Sixth Edition). Amsterdam; Boston: Academic Press.

[50] Fox MD, Snyder AZ, Vincent JL, Corbetta M, Van Essen DC, et al (2005) The human brain is intrinsically organized into dynamic, anticorrelated functional networks. Proceedings of the National Academy of Sciences of the United States of America 102: 9673–9678. 15976020 10.1073/pnas.0504136102 PMC1157105

[51] Zhang Y, Brady M, Smith S (2001) Segmentation of brain MR images through a hidden Markov random field model and the expectation-maximization algorithm. IEEE Trans Med Imaging 20: 45–57. 11293691 10.1109/42.906424

[52] Obrig H, Neufang M, Wenzel R, Kohl M, Steinbrink J, et al (2000) Spontaneous low frequency oscillations of cerebral hemodynamics and metabolism in human adults. NeuroImage 12: 623–639. 11112395 10.1006/nimg.2000.0657

[53] Shipley MT, Ennis M, Puche AC (2004) Chapter 29 - Olfactory System. In: George P, editor. The Rat Nervous System (Third Edition). Burlington: Academic Press. pp. 923–964.

[54] Scremin OU (2004) Chapter 33 - Cerebral Vascular System. In: George P, editor. The Rat Nervous System (Third Edition). Burlington: Academic Press. pp. 1167–1202.

[55] Smith SM, Fox PT, Miller KL, Glahn DC, Fox PM, et al (2009) Correspondence of the brain's functional architecture during activation and rest. Proceedings of the National Academy of Sciences of the United States of America 106: 13040–13045. 19620724 10.1073/pnas.0905267106 PMC2722273

[56] Miller KL, Jezzard P (2008) Modeling SSFP functional MRI contrast in the brain. Magnetic resonance in medicine : official journal of the Society of Magnetic Resonance in Medicine/Society of Magnetic Resonance in Medicine 60: 661–673. 10.1002/mrm.21690 18727099

[57] Kim TS, Lee J, Lee JH, Glover GH, Pauly JM (2012) Analysis of the BOLD characteristics in pass-band bSSFP fMRI. International Journal of Imaging Systems and Technology 22: 23–32. 23661904 10.1002/ima.21296 PMC3646401

[58] Zhong K, Leupold J, Hennig J, Speck O (2007) Systematic investigation of balanced steady-state free precession for functional MRI in the human visual cortex at 3 tesla. Magnetic Resonance in Medicine 57: 67–73. 17191247 10.1002/mrm.21103

[59] Raichle ME, Mintun MA (2006) Brain work and brain imaging. Annual Review of Neuroscience 29: 449–476. 10.1146/annurev.neuro.29.051605.112819 16776593

[60] Golanov EV, Yamamoto S, Reis DJ (1994) Spontaneous waves of cerebral blood-flow associated with a pattern of electrocortical activity. American Journal of Physiology 266: R204–R214. 8304543 10.1152/ajpregu.1994.266.1.R204

[61] Peltier SJ, Noll DC (2002) T(2)(*) dependence of low frequency functional connectivity. Neuroimage 16: 985–992. 12202086 10.1006/nimg.2002.1141

[62] Koopmans PJ, Boyacioglu R, Barth M, Norris DG (2012) Whole brain, high resolution spin-echo resting state fMRI using PINS multiplexing at 7 T. NeuroImage 62: 1939–1946. 22683385 10.1016/j.neuroimage.2012.05.080

[63] Banich MT, Belger A (1990) Interhemispheric interaction: how do the hemispheres divide and conquer a task? Cortex 26: 77–94. 2354647 10.1016/s0010-9452(13)80076-7

[64] Hutton C, Bork A, Josephs O, Deichmann R, Ashburner J, et al (2002) Image distortion correction in fMRI: A quantitative evaluation. Neuroimage 16: 217–240. 11969330 10.1006/nimg.2001.1054

[65] Lee J, Shahram M, Schwartzman A, Pauly JM (2007) Complex data analysis in high-resolution SSFP fMRI. Magnetic Resonance in Medicine 57: 905–917. 17457883 10.1002/mrm.21195

[66] Chappell M, Haberg AK, Kristoffersen A (2011) Balanced steady-state free precession with parallel imaging gives distortion-free fMRI with high temporal resolution. Magnetic Resonance Imaging 29: 1–8. 20864288 10.1016/j.mri.2010.07.007

[67] Patterson S, Beyea S, Bowen CV (2011) FMRI using high flip-angle alternating steady-state balanced SSFP supported by Monte Carlo studies. Proceedings of International Society of Magnetic Resonance in Medicine. pp. 1630.

[68] Albert Kir ABM, Gullapalli R, Morris JM (2013) High resolution frequency-modulated bSSFP fMRI. Proceedings of International Society of Magnetic Resonance in Medicine. Salt Lake City. pp. 2280.

[69] Foxall DL (2002) Frequency-modulated steady-state free precession imaging. Magnetic Resonance in Medicine 48: 502–508. 12210915 10.1002/mrm.10225

